# Spatially weighted functional clustering of river network data

**DOI:** 10.1111/rssc.12082

**Published:** 2014-10-14

**Authors:** R A Haggarty, C A Miller, E M Scott

**Affiliations:** University of GlasgowUK

**Keywords:** Covariance, Functional data, Hierarchical clustering, River networks, Water quality

## Abstract

Incorporating spatial covariance into clustering has previously been considered for functional data to identify groups of functions which are similar across space. However, in the majority of situations that have been considered until now the most appropriate metric has been Euclidean distance. Directed networks present additional challenges in terms of estimating spatial covariance due to their complex structure. Although suitable river network covariance models have been proposed for use with stream distance, where distance is computed along the stream network, these models have not been extended for contexts where the data are functional, as is often the case with environmental data. The paper develops a method of calculating spatial covariance between functions from sites along a river network and applies the measure as a weight within functional hierarchical clustering. Levels of nitrate pollution on the River Tweed in Scotland are considered with the aim of identifying groups of monitoring stations which display similar spatiotemporal characteristics.

## Introduction

Monitoring levels of water pollution has been a key focus of research and legislation in recent years and the introduction of policy such as the European Union Water Framework Directive (European Parliament, 2000) requires that regulatory agencies have extensive monitoring networks in place. These networks are required to ensure that there is a satisfactory quantity of data on which to base classification, and so that any areas which are changing in status can be identified. The spatial selection of monitoring locations as well as the identification of vulnerable areas are key considerations when designing an effective sampling programme with the aim of assessing environmental status. Investigation of similarities in temporal patterns of water quality parameters and spatial correlation across networks can provide information to feed into future monitoring strategies.

In many environmental examples where spatial correlation is considered, Euclidean distance is the natural metric that is used. However, this is not always the most suitable measure of distance. Measurements collected over networks that consist of connected segments are one such situation where the characteristics of the spatial data may be inadequately described in terms of Euclidean distance. River basins are such a case, for which stream distance (the shortest distance between two stations along the stream network) is more suitable.

Straightforward substitution of stream distance instead of Euclidean distance will not always produce a valid spatial covariance model (Ver Hoef *et al*., 2006). Alternative models for use with this type of data have been proposed by Ver Hoef *et al*. (2006) and Cressie *et al*. (2006) and not only incorporate stream distance but also take into account other defining properties of directed river network structures such as the flow connectedness between stations.

Frequently in environmental data contexts the unit of interest is a time varying function. Therefore we propose a functional data analysis approach to define groups of curves which are similar in both seasonal and long-term patterns. Regarding the data as functional not only enables easier identification of common patterns in the data across individual monitoring stations; it has the further advantage of overcoming some of the problems that are associated with irregularly spaced or sparse data, since the curves, and not individual samples, become the objects of interest. Examples are available in the literature where functional clustering has been applied to environmental data (Henderson, 2006; Pastres *et al*., 2011; Ignaccolo *et al*., 2008). In most cases the assumption is made that the individual stations being grouped are spatially independent. Although examples where spatial correlation is present in environmental data are abundant in the literature where non-functional statistical techniques are applied (Guttorp *et al*., 1994; Akita *et al*., 2007), there are fewer examples which discuss the inclusion of spatial correlation within clustering methods, and only a couple which examine the presence of spatial correlation for geographically referenced functional data. Delicado *et al*. (2010) have provided a summary of recent contributions to methods of interpolation for functions with different Euclidean-based spatial data structures. The methods discussed include approaches proposed by Goulard and Voltz (1993), Nerini *et al*. (2010), Giraldo *et al*. (2011) and Menafoglio *et al*. (2013) which all aim to offer a solution to the problem of predicting curves at unsampled locations. However, prediction at monitoring locations is not the objective of this work. Two examples of spatial functional clustering were provided in Romano *et al*. (2010) and Secchi *et al*. (2011), both of whom used iterative algorithms to partition geographically referenced data. In addition, Giraldo *et al*. (2010) extended existing ideas used for clustering to include spatial correlation between curves. Hierarchical clustering methods are adapted via weighting the dissimilarity matrix by a measure of spatial functional covariance. Another recent example of classification of spatially interdependent functional observations is provided in Jiang and Serban (2012), who used a non-parametric model-based method with a spatially correlated error structure to classify service accessibility patterns for the financial services industry.

Although standard spatial statistical tools based on Euclidean distance have been generalized for use with functional data there appear to have been no examples where functional data on a directed network have been explored. The aims of this paper are, firstly, to develop an approach which can be used to estimate the spatial covariance between monitoring stations on a river network when the observations at each of these stations are curves. Secondly, it is our aim to incorporate this estimate of the covariance structure within a hierarchical functional cluster analysis to identify groups of stations which display similar spatiotemporal characteristics and, finally, to apply this approach to a case-study comparing the clusters that are obtained with and without spatial covariance-based weights.

In Section Data we shall discuss a set of water quality data from the River Tweed in Scotland that will be used within our analysis whereas functional hierarchical clustering and the measure of river network spatial covariance that are developed will be discussed in Section Methodology. Section Clustering the tweed data considers the application of spatially weighted hierarchical functional clustering to the River Tweed data with a discussion of the results provided in Section Discussion.

## Data

Concentrations of chemical determinands such as nitrates are often used to assess fresh water quality. Although nitrates stimulate the growth of plankton and other aquatic plant life, their presence in excess can cause eutrophication. The data that are used in the analysis presented in this paper are nitrate concentrations from a network of locations along the River Tweed, located in the Scottish borders, and have been provided by the Scottish Environment Protection Agency. Agriculture is an important industry within the area and the water environment in the wider River Tweed catchment is a substantial economic, social and environmental asset (Scottish Environment Protection Agency, 2009). Data have been provided at 77 unique monitoring stations. The observations are approximately monthly and cover a 9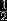-year period from the spring of 1997 to the winter of 2006.

**Figure 1:**
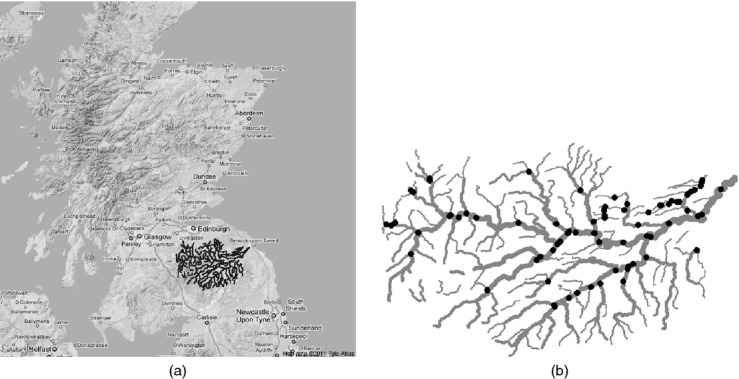
(a) Map of Scotland, northern England and Northern Ireland, showing the location of the River Tweed and (b) the River Tweed network showing the locations of the monitoring stations

The location of the Tweed within Scotland is shown in Fig. 1(a) and the river network itself is shown in Fig. 1(b) where each of the points shown on the network is a monitoring station location. Although the picture shows some apparently unconnected stream segments, this is because small lochs and other types of water body have not been shown. It is clear that the Tweed network has a complex structure with many tributaries flowing into the main stream, which is represented by the bold line which runs from the far south-west to the north-east. In total there are 298 stream segments. Flow data provided by the Scottish Environment Protection Agency were estimated for the Tweed by using a computer package called Low Flows 2000 (Goodwin *et al*., 2004). As the flow data are estimated rather than observed they remain static throughout the time period that is considered.

## Methodology

Hierarchical functional clustering has been used to identify groups of stations where the patterns in nitrate concentrations are similar over time. Hierarchical clustering enables spatial covariance to be easily incorporated in the clusters by weighting the distance matrix by the covariance matrix. However, a method of estimating the spatial correlation is required which can take into account the directed river network structure.

To apply hierarchical clustering to a set of points in the non-functional data setting, a distance matrix *D* is calculated, where the (*i*,*j*)th entry of *D* is the distance between points *i* and *j* as determined by whichever metric has been chosen. Although calculating the distance between pairs of functional objects may seem less straightforward to compute, Henderson (2006) states that the idea of measuring distances is easily transferable from pairs of points to pairs of curves and defines a method of computing a functional distance matrix.

Let there be *N* curves and let *g*_*i*_(*t*) denote the value of the *i*th curve at time *t*. If the curves are estimated by using spline functions then each estimated curve 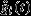 can be expressed as the product of a set of spline coefficients **c**_*i*_ and a vector of basis functions Φ(*t*) as follows:


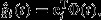


where *i*=1,…,*N*. Let the *i*th and *j*th estimated curves 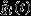 and 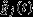 be expressed as a linear combination of basis functions with coefficient vectors **c**_*i*_ and **c**_*j*_ respectively. The distance between the curves can then be written as


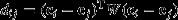
(1)

and *W*=∫Φ(*t*) Φ^T^(*t*) d*t*, which is a symmetric square matrix of order *P*, and *P* is the number of spline basis functions. For each set of basis functions, *W* can be evaluated by using numerical integration, if necessary, and the functional distance matrix *D* with entries *d*_*ij*_, as defined above, can be computed. Standard algorithms for hierarchical clustering can then be applied to the functional distance matrix.

One of the main difficulties that are associated with cluster analysis is determining the most appropriate number of clusters given the data. Several approaches have been proposed in Calinski and Harabasz (1974), Hartigan (1975) and Krzanowski and Lai (1988). One popular approach for selecting the number of clusters is the gap statistic that was proposed by Tibshirani *et al*. (2001) which compares the average within-cluster dispersion for the observed data, with the average within-cluster dispersion for a null reference distribution which assumes that there is no clustering within the monitoring stations. Several reference data sets are simulated and, for each, the same clustering technique that was applied to the observed data is used. These simulated curves are assumed to have no cluster structure as they have been randomly generated. The gap statistic has been used as the method to select the statistically optimal number of clusters throughout this paper.

### Spatially weighted functional clustering

Oliver and Webster (1989) proposed the idea of weighting a distance matrix by a covariance matrix to obtain clusters which take into account spatial correlation. This idea has been extended to the functional clustering case by Giraldo *et al*. (2010) who proposed incorporating spatial covariance in hierarchical functional clustering by weighting the functional distance matrix, defined in equation (1), using a functional covariance matrix that has been estimated by using an appropriate variogram.

The idea of generalizing the variogram to be used with spatially correlated functional data was discussed in Giraldo *et al*. (2011) who proposed the trace variogram. Let *g*_1_(*t*),…,*g*_*N*_(*t*) defined for *t* ∈ [*a*,*b*]⊂**R** be a set of curves which are realizations of a stationary, isotropic functional random process collected from *N* stations over time *t* with corresponding location co-ordinates denoted by *x*_1_,…,*x*_*N*_. Then, writing the distance between two locations (*i*,*j*) as *h*, the trace variogram can be defined as


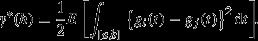
(2)

An estimate of the integral in equation (2) is equivalent to the square of the estimated functional distance since


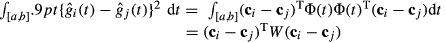


where *W*=∫_[*a*,*b*]_*pt*Φ(*t*) Φ(*t*)^T^ d*t*. As with standard variograms, to obtain the empirical trace variogram, the trace variogram cloud can be computed by calculating the differences between all pairs of curves and plotting these differences against the corresponding distance between the locations. The points on this plot can then be ‘binned’ and averaged at a series of regular intervals. The estimated trace variogram can therefore be written as


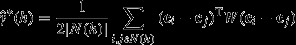
(3)

where |*N*(*h*)| is the number of curves separated by a distance of *h* units. After obtaining the empirical trace variogram from observed data, any standard variogram model can be fitted as if it were a standard univariate variogram. Giraldo *et al*. (2010) noted that the fitted parametric trace variogram is always a valid variogram because its properties are those of a parametric variogram fitted from a univariate geostatistical data set.

The trace variogram method above has been developed with Euclidean distance in mind; however, to estimate a covariance structure for stations on a river network, stream distance, rather than Euclidean distance, is used. Usually when Euclidean distances are used to define spatial covariance a covariogram model can be used, although, when fitting a model to an experimental covariogram based on stream distances, there are some additional issues that need to be addressed. Spherical and linear models used in combination with stream distances can result in a covariance matrix which is not positive definite and is therefore invalid. The tail-up model that was introduced by Ver Hoef *et al*. (2006) is a valid model which can be used in conjunction with stream distances and assigns a covariance of 0 to stream segments which are not flow connected. Given a response of nitrate, the tail-up model is the most appropriate formulation in this example.

To define the tail-up model, let *Z*(*x*_*s*_) and *Z*(*x*_*r*_) be the values of a random variable at locations *x*_*s*_ and *x*_*r*_ which are on stream segments *s* and *r* respectively, and let *h*_S_ be the stream distance between them. Let 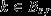, where *B*_*s*,*r*_ is the set of all stream segments on the river network that are between segment *s* and segment *r*. Following this, it was shown in Ver Hoef and Peterson (2010) that a class of tail-up models that is suitable for use with stream distance can be written as


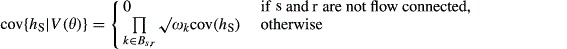
(4)

where cov(*h*_S_) is the standard Euclidean-distance-based model formulation of a chosen covariance function with parameters *θ*, e.g. the Matérn function, and ***ω***_*k*_ refers to a set of weights. From equation (4) it can be seen that the weighting between any two flow-connected points on the river, say *s* and *r*, is obtained by taking the product of the square root of the *k* weights over the set *B*_*s*,*r*_, which corresponds to all stream segments that lie between *s* and *r*. It was suggested by Ver Hoef *et al*. (2006) that the best way in which to define the weights ***ω***_*k*_ is by using flow volume. Including these weights in this way effectively means that covariances between two stations which are flow connected will be relatively small if one of the stations is on a minor (low flow volume) stream segment, even if the two stream segments are close in terms of the stream distance.

### Functional covariance between stations on a river network

Owing to the complex structure of the tail-up model shown in equation (4) it is necessary to model spatial correlation on a river network by using a covariogram. To fit the tail-up model, the first step is to compute the observed pairwise covariances for all pairs of stations which are flow connected. Subsequently, these covariances can be plotted against lags (measured in terms of stream distance) and binned at regular intervals to obtain an empirical stream-distance-based covariogram.

To estimate the stream distance covariance for functional data, we need to define a metric for measuring the covariance between two curves. For standard, non-functional data, Cressie (1993) stated that the estimated covariance between stations *s*_*t*_ and *r*_*t*_ at one particular point in time, *t*, is given by


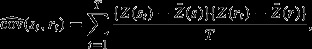
(5)

where *Z*(*s*_*t*_) and *Z*(*r*_*t*_) are the values of the variable at stations *s* and *r* at time point *t* and *T* is the total number of time points. Keeping in mind both the above equation and the definition of the trace variogram in equation (3), which uses the area between two curves to represent the difference between them, one potential measure for estimating functional covariance has been developed. Using the same notation as before, let *g*_1_(*t*),…,*g*_*N*_(*t*) defined for *t* ∈ [*a*,*b*]⊂**R** be a set of *N* curves which are realizations of a stationary, isotropic functional random process collected from *N* stations with corresponding location co-ordinates denoted by *x*_1_,…,*x*_*N*_. Also as before, if each estimated curve is expressed by using *P* basis functions as 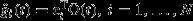, then each set of coefficients 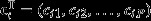 is a vector of length *P*. The functional mean of all *N* curves, 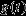, can be defined by the mean of the basis coefficients representing the set on all *N* curves at time point *t*,


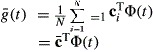


where


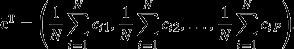


**Figure 2:**
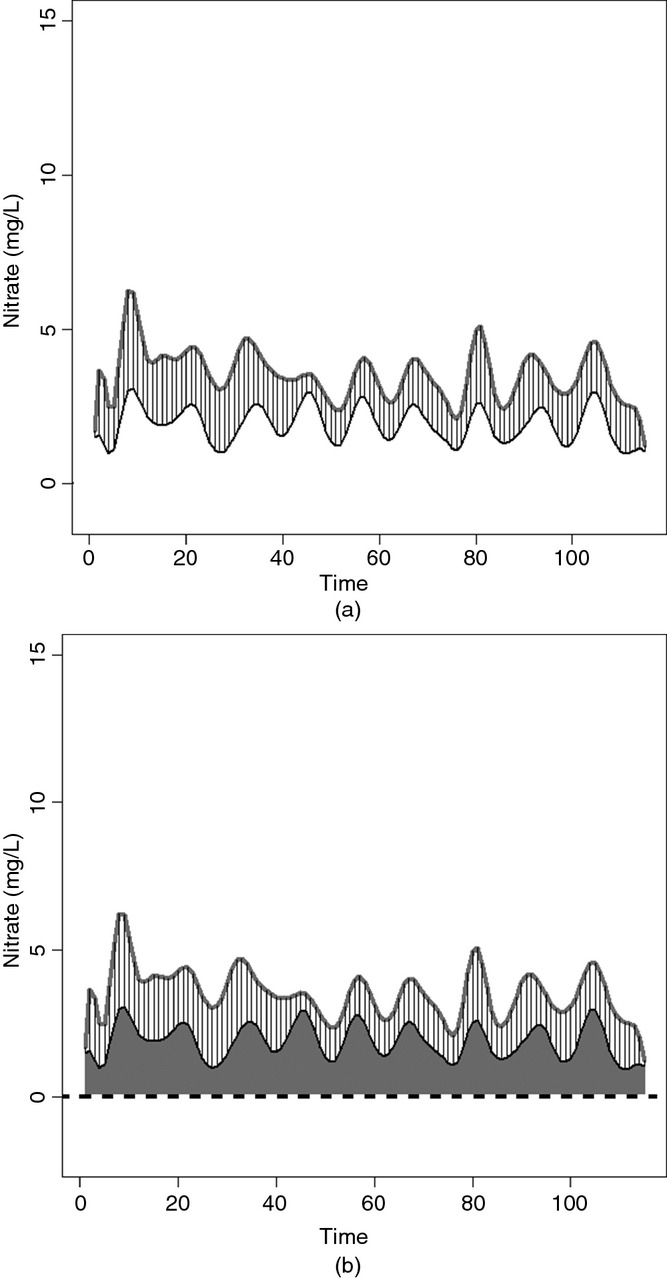
Nitrate concentration at River Tweed station 1 (———) alongside the functional mean nitrate curve for all stations (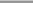), with the reference line (– – – –) (the reference line is shown in (b) only)

One key problem is that multiplication of two areas is likely to result in large values which are not suitable in the computation of covariances and it is thought that the covariance will, at some point, tend to 0 as the distance between stations increases. To overcome this problem standardization of the areas is required; in this case a ‘reference line’ can be defined. The area between the mean curve and this reference line (which is shown by the total of the black lined area and the grey-shaded area in Fig. 2(b)) can be used both to reflect the direction of the difference between a given station and the overall mean and can be used to standardize the areas so that the measures of covariance are on a suitable scale. Since curves should not fall below this reference line it should be set as a horizontal line which is below the minimum value of the set of *N* curves **g**(*t*).

Writing the reference line as *g*_R_ and the corresponding set of basis coefficients which define this line as **c**_R_, then the area between the reference line and the mean curve can be written as


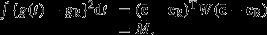


Similarly, the area between the estimated curve representing station *i* and the reference line can be written as


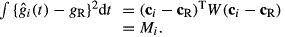
(6)

Then the difference between these two measures, 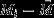, reflects the magnitude of the difference between curve 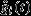 and the mean curve 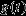 and gives an indication whether the station has higher- or lower-than-average values. This difference can then be standardized by dividing by the difference between the mean curve and the reference line 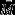, to ensure that the scale is appropriate. For example, in Fig. 2(b), the broken horizontal line represents the reference line *g*_R_, the black lined area represents *M*_1_, the difference between the curve (shown by the black curve) and the mean curve (shown by the grey curve). The reference area *M*_R_ is the area between the mean curve and the reference line.

Following from this, the functional covariance between stations *i* and *j* can be estimated as


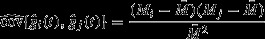
(7)

where *i*≠*j*. This results in a single value which summarizes the covariance between the functions at the two stations over the time period of interest. These point summaries of the covariance between pairs of curves can then be used to create an adjusted covariogram cloud. In accordance with the definition of the tail-up model that is described in equation (4), a standard covariogram model, such as the Matérn function, can next be fitted to this empirical covariogram. Evaluating this model at the relevant stream distances will result in an estimated stream-distance-based functional covariance matrix *V*. To obtain a valid estimated stream-distance-based covariance matrix 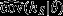, the elementwise product of *V* and the weight matrix can be computed.

Although the selection of the baseline may seem rather arbitrary, it is just one suggested approach for standardizing the covariance estimates. Provided that the reference line is a horizontal line which has a value that is lower than the global minimum value of the curves, the choice of baseline will not affect the covariance measure estimated. Alternative methods of standardization could also be used.

To find spatially homogeneous clusters of stations on a river network, 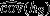 can be used as a weight matrix:


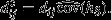
(8)

Here, as before, *d*_*ij*_ is the functional distance matrix (equation (1)).

The key difficulty in clustering stations on a river network is due to the necessity to model the covariance between the stations rather than the semivariance. Defining the covariance between curves in the manner that is outlined in equation (6) enables a summary value of the spatial dependence between stations to be obtained. Consequently, these values can be used to fit the tail-up covariance model which can be used to estimate a valid stream-distance-based covariance structure that takes into account stream distance, flow weights and flow connectedness between stations. These are features of the data which are not considered when using the Euclidean-distance-based trace variogram to cluster river network data.

## Clustering the tweed data

Exploratory analysis of the Tweed nitrate data highlighted that there were substantial differences between the stations in terms of the amplitude of the seasonal patterns that are observed, and in terms of the temporal mean levels. A natural next step was to investigate applying a log-transformation to the data. Although there continued to be a difference between the temporal mean levels at the stations, albeit less distinct than on the original scale, there was less disparity between the strength of the seasonal patterns at the stations. In addition, there remained evidence of non-constant variance at some stations and the log-transform did little to overcome this potential issue in the data. This change in variability was explored further and was found to be a change in the seasonal pattern over time. One aim of our analysis was to obtain groups of stations which are similar in terms of temporal patterns of the determinand of interest, while taking into account any long-term trends and seasonal patterns. After exploratory investigation of the Tweed stations, it was decided that taking the log-transform of the nitrate data often ‘dampened’ features of the data which were thought to be of most interest in distinguishing different groups of stations, such as changes in the seasonal pattern over time. In addition, although there are differences in temporal mean levels of the observed data at different stations, these differences are not particularly extreme and so it seems reasonable to compare the stations without transforming the scale of the data. Consequently, all further analyses on the Tweed data were carried out by using the raw data as it was felt that this would produce more informed groupings for this data set.

Initial investigation of nitrate levels across the geographical region that is covered by the Tweed network also indicated that it was unlikely that the assumption of spatial stationarity holds. Hence it was necessary to remove the long-term spatial trend in nitrate levels across the network before estimating the covariance structure. It was also clear that a parametric trend would not be adequate to describe the spatial patterns in the data so a simple non-parametric trend was estimated by using the mgcv library in R (Wood, 2003). Using this package, a bivariate smooth trend was fitted to the temporal station means by using thin plate regression splines with a smoothing penalty applied to ensure that the estimate of the spatial trend retained the key features of the data without being overly sensitive to small changes. Time series means at each station were used as the response to provide a broad estimate of the spatial trend over the time period of interest. As one of the primary aims of this analysis was to cluster curves in the presence of spatial correlation, and not to model the correlation structure explicitly or to predict at unsampled locations, it was felt that this approach for removing the spatial trend in the data was sufficient. Stationarity appeared to hold when considering the residuals of the Tweed nitrate data after removing the spatial trend in this way.

Functional data were obtained by fitting curves to the spatially detrended nitrate data at each of the stations using a penalized regression spline approach. A second-order roughness penalty term was also used within the least squares estimation of the spline coefficients to ensure that the curves reflected the underlying pattern in the data accurately without being too locally variable. Both the number of spline basis functions and the smoothing parameter which controls the effect of the penalty term were selected by using a sensitivity analysis. Using a graphical approach to assess an appropriate degree of smoothing which captured the trend and seasonal patterns over time in the data, the number of spline basis functions chosen was 36 and the smoothing parameter was selected to be 0.01. On the basis of the sensitivity analysis the smoothing parameter was found to ensure that the curves representing nitrate concentrations on the original scale were positive. If necessary, the form of the spline could be tailored to the specific context for any required constraints. To ensure that the number of clusters that are identified by using the gap statistic was not overly sensitive to the smoothing parameter selected the gap statistic was run a number of times, each time for a set of curves fitted with a different degree of smoothing. Within a reasonable range of smoothing parameter values the gap statistic consistently identified the same number of clusters as optimal each time.

The empirical functional covariogram was estimated using the functional covariances calculated by using equation (7) and the stream distance between the stations (measured in kilometres). The points were ‘binned’ at 10-km intervals, with an additional point estimated at 5 km since many of the stations were separated by short lags. The covariogram was estimated up to a maximum distance of 70 km since over 90% of the distances were shorter than this distance. A Matérn covariance function was fitted to the empirical covariogram by using weighted least squares. The Matérn covariance structure was used as it is thought to be very flexible and provides a general set of functions which encompasses several of the covariance functions that are commonly used to estimate the spatial covariance of environmental data. Weighted least squares, with the weights as defined in Cressie (1985), were used as the method for selecting the optimal covariance parameter values. This approach of estimating the covariogram was selected as it was computationally efficient to apply and performed well at capturing the pattern in the observed data. Other methods such as maximum likelihood could also be used to estimate the covariogram.

**Figure 3:**
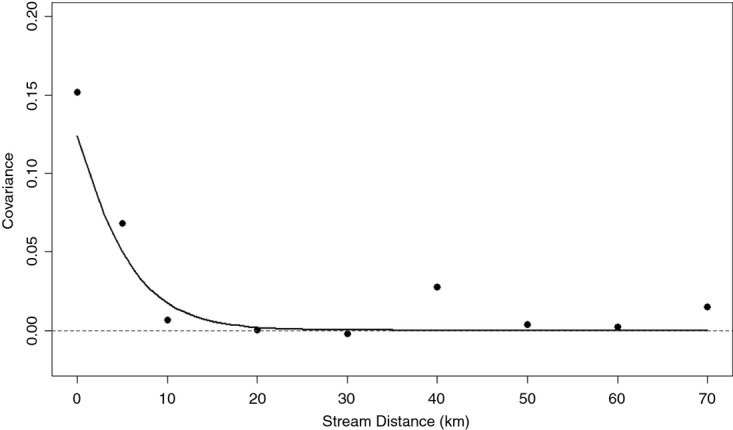
Estimated (•) and fitted Matérn (———) covariograms for the (spatially detrended) Tweed nitrate data

Fig. 3 shows the functional covariogram for the Tweed nitrate data with the fitted Matérn covariance function shown by the full curve. As the Matérn family covariance functions have an infinite range and approach the sill asymptotically, the effective range *h*_0.95_ as defined in Cressie (1993) has been calculated. This is the distance which corresponds to 5% of the maximum covariance. For stream distance, the covariogram fitted indicates that, after spatially detrending the data, flow-connected stations are spatially correlated until they are separated by a distance of more than 15.4 km. Using the estimated covariogram, a covariance matrix was obtained. In line with equation (4), to ensure that the covariance matrix was valid, the stream distance matrices obtained were multiplied through by the square root of the flow weights. The covariance matrix that was obtained by using the functional covariogram was then used to weight the functional dissimilarity matrix.

After estimating the covariance structure on the basis of the spatially detrended functions hierarchical clustering was applied to the original set of smoothed curves. Clustering of the Tweed data was considered for three covariance scenarios:

1. spatially independent;
2. Euclidean-distance-based spatial covariance;
3. stream-distance-based spatial covariance.

Although the trace variogram can be used to estimate the Euclidean-distance-based covariance structure, the functional covariogram approach that was described above was used. This was done to ensure that results obtained by using each of the Euclidean and stream distance covariance weighted clusterings of the stations were comparable.

For the hierarchical clustering which assumes spatial independence, the statistically optimal number of clusters determined by using the gap statistic with 500 reference distributions was 7. For the Tweed data, the null reference distribution that was used within calculation of the gap statistic was generated on the basis of observed data. The new data were randomly generated by using a relevant uniform distribution constrained between the maximum and the minimum values of the observed data. Data were generated to represent a set of curves (the same number of curves as in the observed data set). When spatial covariance was incorporated, fewer groups were identified as being sufficient to capture the differences in the nitrate concentrations among the stations adequately: five for Euclidean distance covariance weighted clustering and just three clusters for stream distance covariance weighted clustering. The results of the clustering approaches are summarized in Figs 6 which display maps showing the clusters and corresponding plots of the cluster mean curves. On each of the cluster mean plots, the black broken curve represents the overall mean for all stations.

**Figure 4:**
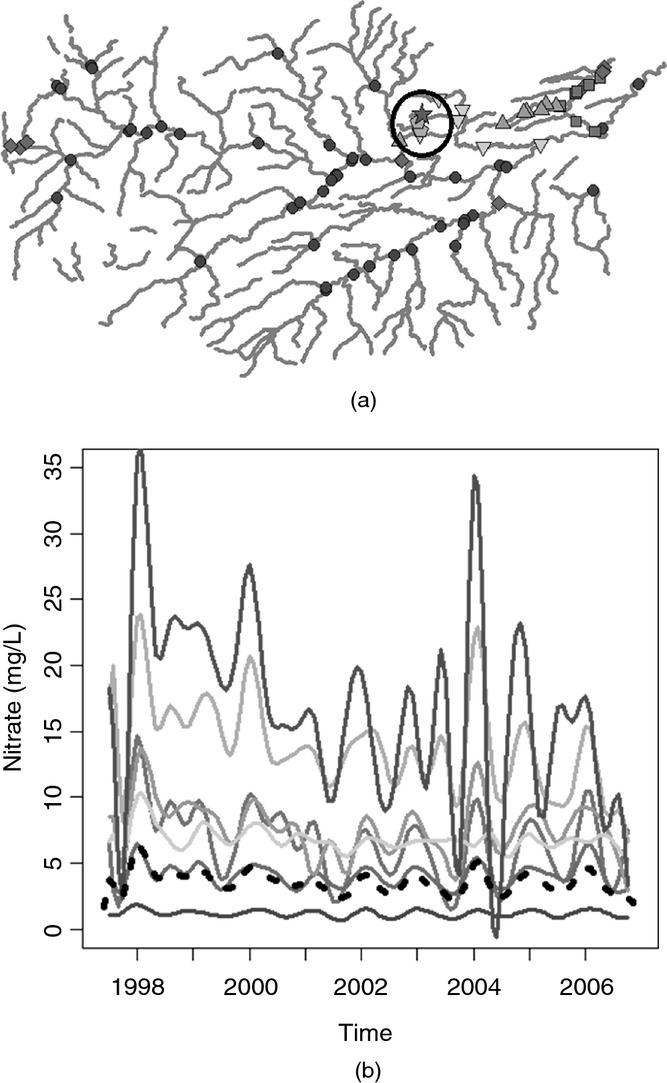
Hierarchical clustering results by assuming spatial independence: (a) Tweed network showing various groups; (b) group mean curves (– – –, overall mean curve)

**Figure 5:**
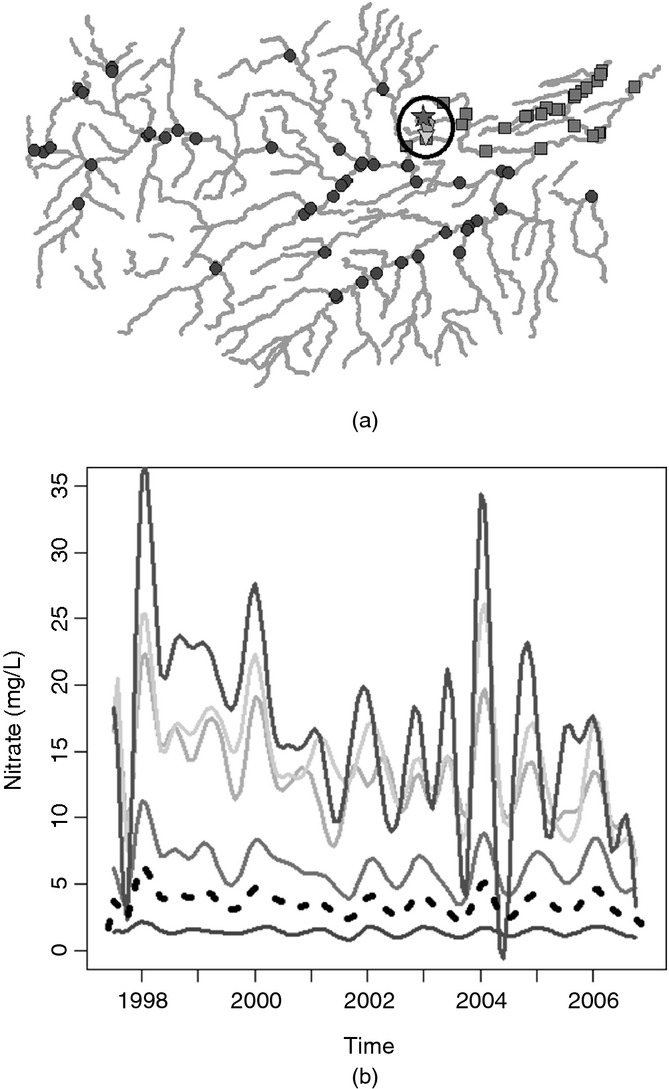
Detrended data Euclidean distance covariance weighted hierarchical clustering results: (a) Tweed network showing various groups; (b) group mean curves (– – –, overall mean curve)

**Figure 6:**
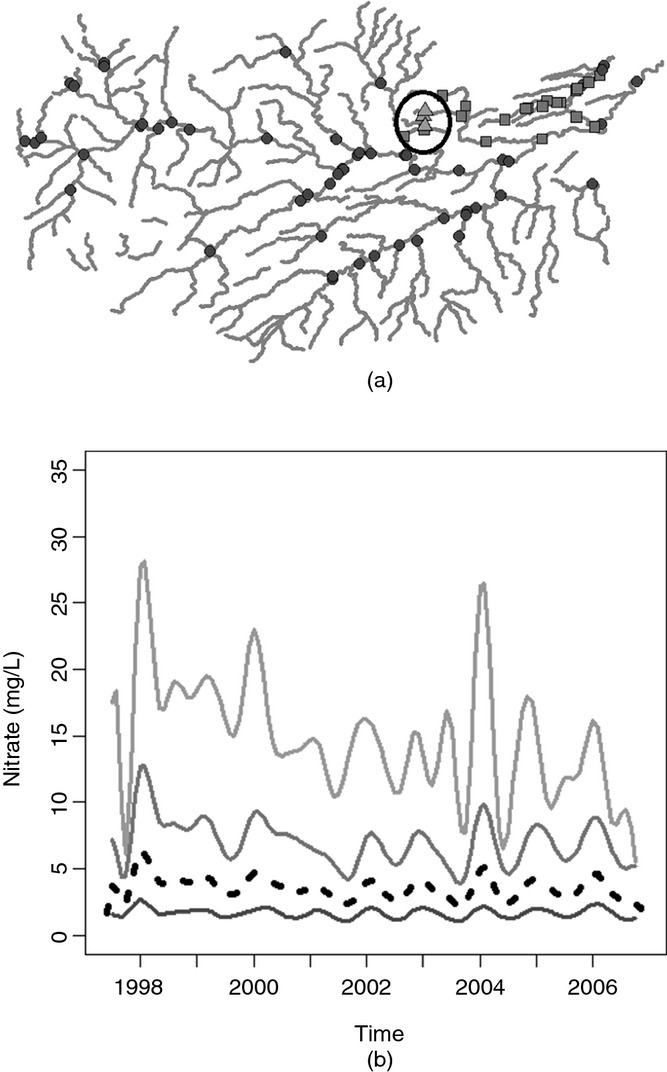
Detrended data stream distance covariance weighted hierarchical clustering results: (a) Tweed network showing various groups; (b) group mean curves (– – –, overall mean curve)

All three sets of clusters identified an area in the north-east of the region (indicated by the circled area in Figs 6) in which stations have a markedly higher mean concentration as well as a much higher seasonal pattern than the other stations. The number of clusters of stations that is identified within this area is where the methods differ. The stream distance covariance weighted clustering identified only one group of stations whereas Euclidean distance clustering identified three groups consisting of either one or two stations each. For the clusters that incorporate the effects of stream covariance it can be seen from Fig. 6 that there is one group of low concentration, low seasonal signal stations, one group of moderate concentration, moderate seasonal signal stations and one group of higher concentration, higher seasonal signal stations. Overall, the cluster means from the Euclidean distance covariance weighted clustering and the spatially independent functional clustering also display a similar split in the group means based on mean level; however, there is an increased number of groups in comparison with the stream distance clustering.

It could be expected that the clusters are more spatially coherent when spatial correlation is taken into account. There are several reasons why this could be expected, one of the most notable of which is land use. Locations that are close in space are more likely to be surrounded by land which is used for the same purposes and this is a key factor when the determinand of interest is nitrate as agricultural run-off is likely to be the source of the majority of nitrate in the water. The increase in coherence in the clusters can be seen when both Euclidean and stream distance covariance are incorporated as both measures will, to some extent, account for unmeasured variables, such as land use effects. The further reduction in the number of clusters when including stream-distance-based covariance may be explained by the incorporation of flow connectedness. Taking into account flow connectedness between stations will affect the spatial coherence of the clusters as only stations which are flow connected will be included in the covariogram estimate, and subsequently in the covariance-matrix-weighted distance matrix.

To quantify how similar the results of each method were the adjusted Rand index (ARI) has been computed (Hubert and Arabie, 1985). The maximum value of the ARI is 1, which corresponds to perfect agreement between two sets of clusters; conversely, if the ARI is 0, the two sets of clusters are mutually independent. There is reasonably strong agreement between hierarchical partitions of the stations by using Euclidean and stream covariance (ARI 0.71). There is slightly less agreement between the spatially weighted clusters and the hierarchical clusters which assumed independence where the ARIs are 0.61 (Euclidean) and 0.64 (stream).

## Discussion

A methodology which incorporates river network covariance in spatially weighted functional cluster analysis has been proposed. The aim has been to identify groups of stations along a river network which are spatially coherent and take into account some of the unique characteristics of a directed river network structure. There is no correct answer about the optimal number of clusters for describing the Tweed data. It is hoped that functional clustering will provide us with more ecologically relevant information than a set of groups obtained solely by clustering the temporal mean at each station. This appears to be the case in the Tweed example as groups are formed not only on the basis of heterogeneity of the temporal mean level, but also on the basis of seasonality. Furthermore, the inclusion of spatial information, both Euclidean and stream distance based, is also informative and may account for unmeasured variables such as land use.

Although Euclidean distance is commonly the most appropriate metric for estimating underlying spatial variability, stream distance is a natural choice when estimating the spatial variation across a network of stations which are linked by a connected river network. However, the structure of a directed network adds complexity to the estimation of spatial covariance and to ensure reliable inference it is necessary to use stream distance and an appropriate covariance model, such as the tail-up model. Developing the standard measure of covariance and ideas from the trace variogram, an estimated functional covariogram has been proposed which uses areas between pairs of curves. The functional covariogram approach enables specific river network features such as stream distance, flow weights and connectedness to be included in the estimation of a spatial covariance structure.

The results from the application of our approach to pollutant data from stations along the River Tweed highlighted the effect of including network respecting spatial covariation when clustering pollutant curves at the stations. In this particular example the tail-up model is most appropriate as the determinand of interest is nitrate concentrations. In other contexts where this is not so there is no reason why the same methodology could not be transferred to other stream distance covariance models such as the tail-down model or variance component models (Ver Hoef and Peterson, 2010). Furthermore, although hierarchical clustering with complete linkage approach or linkage criteria could be substituted. Functional cluster analysis of the River Tweed data by using the methods described along with divisive hierarchical clustering (Kaufman and Rousseeuw, 1990) showed results that are consistent with those presented. Further results and details on this analysis are available in the on-line supplementary materials.

Using the gap statistic, a much smaller number of clusters is identified as statistically optimal when stream-distance-based covariance is incorporated in the clustering relatively to when spatial independence is assumed. Although there is no single correct number for how many groups is best, the stream distance covariance weighted clusters were all distinct in terms of their cluster means. This was in contrast with the clusters which were formed on the assumption of no spatial association between the stations. A similar underlying spatial pattern in the groups was identified by using both approaches, with a high concentration nitrate level in the north-east of the Tweed network; however, there was a far greater overlap in the cluster means when stream distance was not taken into account. This suggests that methods which take into account the spatial correlation perform well in terms of identifying similar groups in cluster analysis of spatially referenced samples of curves. It could naturally be expected that stations that are close have similarities, possibly due to the effects of unmeasured local covariates.

Although the Euclidean and stream distance covariance estimates are based on different sets of monitoring stations owing to the inclusion of the network connectedness in the stream distance case, the effect of changing the sampling stations on the covariance estimation and subsequent cluster structure that is obtained is an interesting question which has not been specifically explored here. This is an area which is subject to future work and is potentially something which could be incorporated in the design of future monitoring networks. The focus of this application is functional clustering of stations on a river network although other general applications could consider estimating covariances for any directed network structure. Transport and service networks may provide other opportunities for the application of the techniques that are discussed here.

## Supporting Information

Additional ‘supporting information’ may be found in the on-line version of this article: ‘Spatially weighted functional clustering of river network data supplementary material’.
