# Supplementary material for: Spatially weighted functional clustering of river network data
Source: J R Stat Soc Ser C Appl Stat. 2014 Oct 14;64(3):491–506. doi: 10.1111/rssc.12082 (PMC4407953; doi:10.1111/rssc.12082)
Supplement: Supplementary file 1 [file rssc0064-0491-sd1.pdf]

Spatially Weighted Functional Clustering of River Network Data

Supplementary Material

R. A. Haggarty* (ruth.haggarty@glasgow.ac.uk)

C. A. Miller (claire.miller@glasgow.ac.uk)

E. M. Scott (marian.scott@glasgow.ac.uk)

School of Mathematics and Statistics, University of Glasgow,

15 University Gardens, Glasgow, G12 8QW

July 18, 2014

Although agglomerative hierarchical clustering with complete linkage was selected, the general methodology proposed could be applied to any distance matrix based clustering. The Tweed data described in the manuscript was also analysed using divisive hierarchical clustering (Kaufman, 2005) to check the robustness of

	**No Spatial**	**Euclidean Distance Covariance Weights**	**Stream Distance Covariance Weights**
Agglomerative	7	6	3
Divisive	8	6	3
ARI coefficient comparing agglomerative and divisive clusters	0.79	0.78	0.85

Table 1: Table showing comparison of number of clusters obtained using hierarchical and divisive clustering of River Tweed data

the approach under different distance matrix based clustering methods. Table 1 contains the numbers of clusters as determined by the gap statistic under each of the clustering approaches and the corresponding Adjusted Rand Index (ARI) value. As can be seen, the results obtained for the divisive clustering, in terms of the number of clusters identified as statistically optimal, were consistent with the agglomerative hierarchical results presented in the manuscript. Further to this, the high ARI values confirm that there is a strong degree of agreement between the partitions obtained under each approach.

Maps showing results of the clusters based on divisive hierarchical clustering are shown in Figure 1, 2 and 3. In these plots different coloured points are used to represent the different clusters and cluster means. The solid black line on the

cluster means plot corresponds to the overall functional mean of the data. It is clear the same key structure in the clusters is identified using both the divisive and hierarchical clustering approaches.

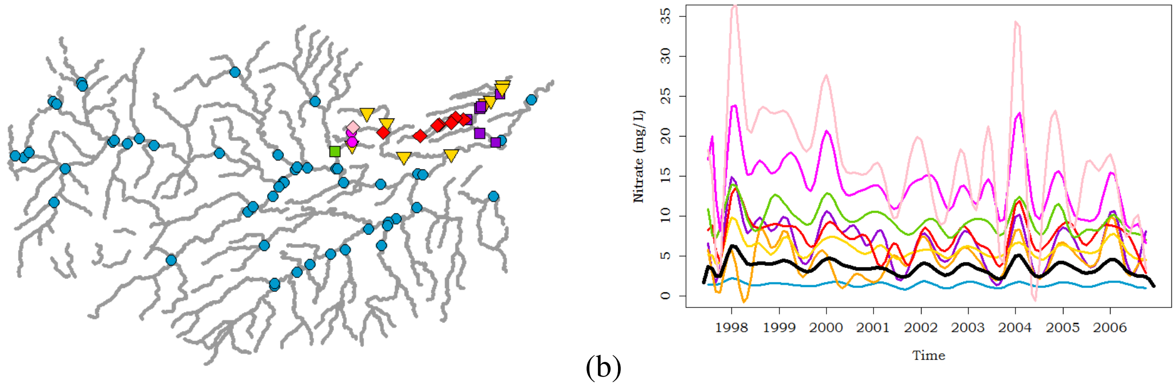

(a) (b)

Figure 1: Plots showing divisive clustering results assuming spatial independence, (a) Tweed network showing different groups, (b) Group mean curves

In addition, standard clustering of the data, with hierarchical clustering of the time series means only was explored.

Spatially detrended time-series station means

For hierarchical clustering, both with and without the incorporation of distance based covariance weights, the gap statistic indicated that there was no clustering structure present in the data when considering spatially de-trended time series means only. This is due to the fact there is one large group of stations with very

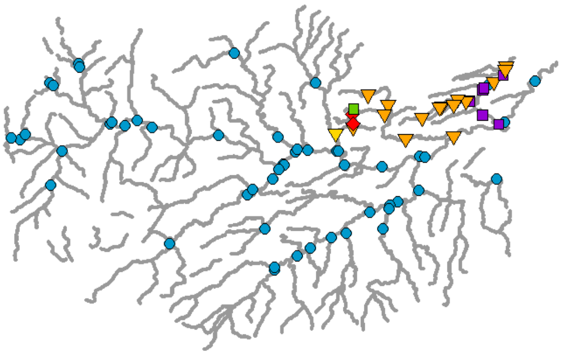

(a)

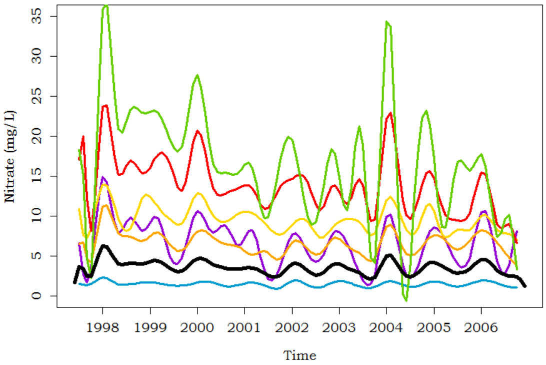

(b)

Figure 2: Plots showing de-trended data Euclidean distance covariance weighted divisive clustering results, (a) Tweed network showing different groups, (b) Group mean curves

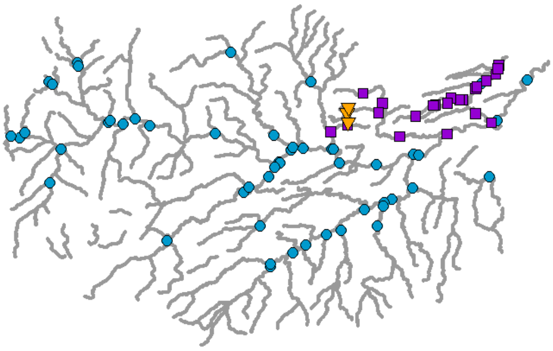

(a)

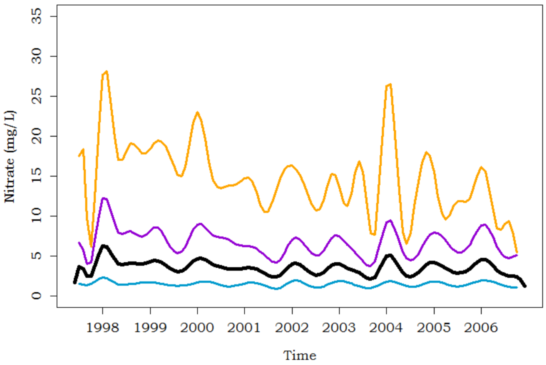

(b)

Figure 3: Plots showing de-trended data stream distance covariance weighted divisive clustering results, (a) Tweed network showing different groups, (b) Group mean curves

similar

temporal means and 3 temporal station means which could be viewed as outliers or singleton clusters. The L-curves were uninformative and the within cluster sum of squares decayed smoothly, indicating no single number of clusters as being optimal.

Original scale station means

The analysis of the standard clustering applied only to mean levels of the time series which did not have the spatial trend removed suggested that 4 groups were required to describe the cluster structure in the data when no spatial variance was incorporated. While it is clear that in this example the clusters are underpinned by the spatial trend in the data, the seasonal patterns at each station also play an important role in distinguishing between groups of sites. This is apparent from the clear cluster structure that is identified using functional clustering, even when the nitrate curves at each station have the estimated spatial mean value removed.

Summary of alternative approaches considered

The application of divisive hierarchical clustering to the Tweed example and the investigation into clusters based on temporal means only has highlighted that the results are robust to different approaches and consistent results are obtained with

all clustering approaches considered. The functional clustering approach presented in the manuscript is thought to be the most informative as it not only incorporates the temporal patterns into the clusters obtained, but can also take into account additional spatial information when weighted by spatial covariance.

Both Euclidean and stream based distance add additional information to the estimated cluster structure, which could be thought of as a proxy for unmeasured variables such as land use. Furthermore, the incorporation of connectedness and flow weights into the stream distance covariance weighted estimates ensures that the covariance estimates obtained are based only on stations which flow into one another, again increasing the information used to find any underlying cluster structure in the data.
